# Experiences from Ukraine in expanding TB infection diagnosis and treatment, including for drug-resistant TB

**DOI:** 10.5588/ijtldopen.25.0670

**Published:** 2026-04-13

**Authors:** V. Shukatka, L. Skoklyuk, M. Germanovych, A. Bogdanov, T. Ivanenko, N. Zherebko, E. Klinkenberg, G. Dravniece

**Affiliations:** 1 PATH, Kyiv, Ukraine;; 2 ConnectTB, The Hague, the Netherlands.

**Keywords:** tuberculosis, TB infection, TBI, QuantiFERON-TB Gold (QFT), interferon-gamma release assay (IGRA), TPT, drug-resistant TB

## Abstract

**BACKGROUND:**

Ukraine faces intersecting epidemics of HIV and drug-resistant TB (DR-TB), with high risk of progression from TB infection to active disease. The Support TB Control Efforts in Ukraine project supported the National TB Program’s efforts to operationalise World Health Organization recommendations on TB infection testing and TB preventive treatment (TPT) across 17 regions of Ukraine.

**DESIGN:**

In September 2021, QuantiFERON-TB Gold Plus (QFT-Plus) testing was introduced through a public–private laboratory model. Eligible populations included TB contacts and other high-risk groups. Data on referrals, test outcomes, and TPT initiation were collected electronically and analysed descriptively.

**RESULTS:**

Out of 11,495 individuals referred for testing, 9,788 (85.2%) completed QFT-Plus testing, with adult TB contacts showing the highest positivity rate at 28.1%. Among 1,717 eligible for TPT, 1,512 (88.1%) started TPT, and this was 93.3% for drug-susceptible TB (DS-TB) contacts and 82.8% for DR-TB contacts. DS-TB contacts were offered shorter rifapentine-based regimens, while DR-TB contacts received levofloxacin. Implementation challenges were addressed through training and mentorship.

**CONCLUSION:**

Ukraine’s implementation of QFT-Plus testing and shorter TPT regimens marks progress in broadening TB prevention efforts, particularly for DR-TB contacts. These initial results highlight that such approaches are feasible even in challenging, high-burden environments, offering insights for future expansion.

TB infection (TBI), defined as a sustained immune response to *Mycobacterium tuberculosis* antigens in the absence of clinical or radiological evidence of active TB disease, affects nearly one-quarter of the global population.^1^ Without treatment, approximately 5%–10% of individuals with TBI will develop active TB disease over their lifetime.^1,2^ Ukraine remains a high-burden country for multidrug-resistant TB (MDR-TB).^3^ In the first 8 months of 2025, 10,667 new TB patients were diagnosed, of whom 2,023 (18.9%) had drug-resistant TB (DR-TB).^2^ TB preventive treatment (TPT) is critical to reduce risk of disease progression and prevent further transmission, particularly in high-risk populations.^4–6^ Since 2020, Ukraine’s National Tuberculosis Program (NTP) incorporated TBI diagnosis and TPT into its strategic framework. National guidelines support the use of modern diagnostic tools – such as interferon-gamma release assays (IGRAs) – and endorse shorter, more effective TPT regimens.

Historically, the tuberculin skin test (TST) was the primary tool to diagnose TBI in Ukraine. While inexpensive and easy to administer, TST has important limitations – especially in Bacillus Calmette-Guérin (BCG)-vaccinated populations – due to reduced specificity and the risk of both false-positive and false-negative results.^7^ Additionally, existing screening practices and logistical considerations influenced testing patterns, which were not always uniformly focused on populations at highest risk, such as household contacts of TB patients. IGRAs, including QuantiFERON-TB Gold Plus (QFT-Plus), have been recommended by the World Health Organization (WHO) since 2011.^8^ Ukraine officially recognised the value of IGRA testing in its national policy in 2014. However, the test was available in only two public health laboratories and only intermittently, while access through commercial laboratories required out-of-pocket payment. Although Ukraine’s TB guidelines endorsed shorter and DR-TB-specific TPT regimens since 2021, they have not been systematically implemented in routine clinical practice. Many health facilities are still in the process of adopting these interventions, highlighting the need for practical tools, provider training, and programmatic support to ensure consistent implementation.

The Support TB Control Efforts in Ukraine (STBCEU) project, funded by the United States Government and led by PATH, assisted the NTP in implementing national TBI diagnosis and prevention policies. Starting in 2021, the project aimed to improve access to IGRA testing, integrate its use into clinical practice, support clinical decision-making based on IGRA test results, promote uptake of shorter and DR-TB-specific TPT regimens, and build the capacity of health care providers to effectively manage TBI. This paper presents Ukraine’s experience and outcomes in expanding TBI testing and uptake of TPT for both drug-susceptible TB (DS-TB) and DR-TB, in alignment with WHO recommendations and national policy.

## METHODS

Implementation began in September 2021 in 12 regions and expanded to 5 more in September 2022. The intervention primarily targeted household contacts of bacteriologically confirmed pulmonary TB patients. The project used age groupings defined by national programmatic practice (0–4, 5–14, 15–17, and ≥18 years), which correspond to the clinical management pathways used in Ukraine. Before TBI testing, active TB was excluded through symptom screening and chest X-ray. Those without evidence of active disease were eligible for TBI testing (Supplementary Data Figure S1). Individuals in whom active TB was diagnosed were referred to start TB treatment as per national guidelines.

### IGRA testing and laboratory network

The QFT-Plus assay was selected as it was the only IGRA method available in Ukraine. An IGRA was made available as an additional option alongside TST. We focused our analysis on individuals tested using IGRAs. Before implementation, an assessment of the laboratory network identified two private laboratories capable of performing QFT-Plus testing with reliable capacity, adequate geographic coverage, and established networks of collection points. Both laboratories were ISO-accredited (ISO 15189:2022) and contracted by the project to provide testing free of charge to individuals, including the supply of consumables and sample transportation. Laboratories submitted monthly reports of completed valid tests and were reimbursed on a per-test basis according to the contracted rate, ensuring payment only for delivered services and service continuity. In two regions without private laboratory collection points, blood samples were collected by TB facility staff and then transported to the contracted laboratories for testing. In these cases, the laboratories supplied the required consumables (e.g., QFT tubes and packaging materials) and organised transportation.

### Clinical coordination

Regional TB physicians or paediatric TB specialists served as coordinators. Their responsibilities included identifying eligible individuals, ensuring pre-test screening, coordinating QFT-Plus testing, reviewing results, and supporting prescription and follow-up of individuals on TPT. Patient registration and test results were captured through private laboratory electronic platforms, which coordinators used for clinical decision-making.

### Clinical protocols and training

A standardised clinical protocol was developed in line with WHO and national guidelines.^9,10^ It addressed identification of risk groups, exclusion of active TB, interpretation of QFT-Plus results, and prescription of TPT. Training activities for regional TB specialists included orientation on national and WHO guidance and practical sessions on blood collection, sample transport, results interpretation, as well as clinical management of TBI and use of shorter TPT regimens. Additional support to regions was provided through mentorship, workshops, and distribution of reference materials. Individuals initiated on TPT were monitored by a medical specialist throughout treatment and were invited monthly for clinical examinations and basic laboratory assessments to detect potential adverse reactions.

### TB preventive treatment

A positive QFT-Plus result was defined as an interferon-gamma level in the antigen tube of ≥0.35 IU/mL.^11^ All individuals with a positive QFT-Plus result were assessed for treatment eligibility, and regimens were prescribed in accordance with national and global protocols.^9,10^ For DS-TB contacts, shorter regimens such as 1HP (1 month of isoniazid and rifapentine), 3HP (3 months of isoniazid and rifapentine), 3HR (3 months of isoniazid and rifampicin), and 4R (4 months of rifampicin) were available. For DR-TB contacts a 6-month levofloxacin regimen (6Lfx) was used. No TPT regimen was available for contacts of pre-extensively drug-resistant/extensively drug-resistant TB (pre-XDR/XDR-TB).^12^

### Monitoring, data collection, and analysis

Implementation was tracked using a standardised electronic reporting system. Standardised data-collection tables were developed for all participating regions and completed by regional coordinators monthly using routinely gathered programmatic data. This included individual-level data on demographics, risk group classification, QFT-Plus results, drug-resistance profile of the index patient, and TPT initiation. Monthly data were reviewed, validated, and checked for completeness and consistency by the project team. Any discrepancies were discussed with regions and corrected. Descriptive analysis was done to monitor uptake and identify operational challenges, informing further training and support.

For the analysis described here, data from October 2021 to December 2024 were included. Summaries of implementation outcomes were generated, and population characteristics, resistance profile, and geographic distribution were described using proportions for binary and categorical variables.

### Ethical statement

This analysis used routinely collected programmatic data from the National Tuberculosis Program, implemented in accordance with national guidelines and WHO recommendations. All diagnostic and preventive interventions were part of standard of care as defined by the Ministry of Health of Ukraine. No experimental procedures were conducted. No personal identifiers was collected, and the data were analysed in an aggregated form to ensure confidentiality.

## RESULTS

Between October 2021 and December 2024, a total of 11,495 individuals were referred for QFT-Plus testing across the 17 regions (Table 1). The demographic profile reflected the programme’s initial prioritisation of child contacts: children aged 5–14 years constituted the majority, 51.7% of all individuals tested. As implementation expanded, however, testing broadened beyond the paediatric focus. Referral numbers increased steadily across all age groups, with adults showing the largest proportional growth – rising from 6.3% of all referrals in late 2021 to 34.5% in 2024.

**Table 1.** Characteristics of the population referred for TB infection testing with QFT-Plus in 17 regions of Ukraine, period October 2021–December 2024 (n = 11,495).

	2021		2022		2023		2024		Overall	
	Oct–Dec		Jan–Dec		Jan–Dec		Jan–Dec		Oct 2021–Dec 2024	
	N = 1,084		N = 2,773		N = 3,724		N = 3,914		N = 11,495	
Age group (years)										
0–4 years	210	19.4%	422	15.2%	391	10.5%	336	8.6%	1,359	11.8%
5–14 years	683	63.0%	1,550	55.9%	1,913	51.4%	1,802	46.0%	5,948	51.7%
15–17 years	123	11.3%	246	8.9%	386	10.4%	424	10.8%	1,179	10.3%
>18 years	68	6.3%	555	20.0%	1,034	27.8%	1,352	34.5%	3,009	26.2%
Sex										
Female	517	47.7%	1,501	54.1%	1,995	53.6%	2,103	53.7%	6,116	53.2%
Male	567	52.3%	1,272	45.9%	1,729	46.4%	1,811	46.3%	5,379	46.8%
Risk group										
TB contact – child <5 years	183	16.9%	382	13.8%	302	8.1%	245	6.3%	1,112	9.7%
TB contact – child 5–14 years	590	54.4%	1,321	47.6%	1,385	37.2%	1,339	34.2%	4,635	40.3%
TB contact – adolescent (15–17 years)	118	10.9%	206	7.4%	332	8.9%	335	8.6%	991	8.6%
TB contact – 18+ years	50	4.6%	415	15.0%	669	18.0%	1,008	25.8%	2,142	18.6%
Medical worker	8	0.7%	62	2.2%	188	5.0%	82	2.1%	340	3.0%
Individuals with other reasons for examination	135	12.5%	387	14.0%	848	22.8%	905	23.1%	2,275	19.8%
Resistance profile index patient (for TB contacts only)										
DS-TB	540	57.4%	1,378	59.3%	1,888	70.2%	2,307	78.8%	6,113	68.8%
H monoresistance	11	1.2%	65	2.8%	38	1.4%	43	1.5%	157	1.8%
RR/MDR-TB	292	31.0%	635	27.3%	515	19.2%	420	14.3%	1,862	21.0%
Pre-XDR/XDR	50	5.3%	166	7.1%	139	5.2%	82	2.8%	437	4.9%
Unknown profile	48	5.1%	80	3.4%	108	4.0%	75	2.6%	311	3.5%
Region (Oblast)										
Cherkasy	102	9.4%	159	5.7%	191	5.1%	138	3.5%	590	5.1%
Chernihiv	80	7.4%	119	4.3%	212	5.7%	199	5.1%	610	5.3%
Chernivtsi	–	0.0%	41	1.5%	261	7.0%	375	9.6%	677	5.9%
Dnipro	218	20.1%	669	24.1%	–	0.0%	–	0.0%	887	7.7%
Donetsk	104	9.6%	26	0.9%	–	0.0%	–	0.0%	130	1.1%
Kherson	44	4.1%	52	1.9%	–	0.0%	–	0.0%	96	0.8%
Kirovohrad	50	4.6%	311	11.2%	442	11.9%	360	9.2%	1,163	10.1%
Kyiv	54	5.0%	54	1.9%	145	3.9%	255	6.5%	508	4.4%
Lviv	54	5.0%	319	11.5%	343	9.2%	360	9.2%	1,076	9.4%
Mykolaiv	47	4.3%	24	0.9%	39	1.0%	81	2.1%	191	1.7%
Odesa	195	18.0%	693	25.0%	786	21.1%	727	18.6%	2,401	20.9%
Poltava	52	4.8%	155	5.6%	357	9.6%	343	8.8%	907	7.9%
Rivne	–	0.0%	66	2.4%	248	6.7%	309	7.9%	623	5.4%
Vinnytsia	–	0.0%	28	1.0%	81	2.2%	77	2.0%	186	1.6%
Volyn	–	0.0%	–	0.0%	450	12.1%	587	15.0%	1,037	9.0%
Zakarpattia	–	0.0%	30	1.1%	169	4.5%	103	2.6%	302	2.6%
Zaporizhzhia	84	7.7%	27	1.0%	–	0.0%	–	0.0%	111	1.0%

For the proportions of the resistance profile of the index case, we did not take into account those without a confirmed contact, as they do not have a defined index case.

DS-TB = drug-susceptible TB; H monoresistance = isoniazid-resistant TB; MDR-TB = multidrug-resistant TB; pre-XDR/XDR = pre-extensively drug-resistant/extensively drug-resistant TB; QFT-Plus = QuantiFERON-TB Gold Plus test; RR-TB = rifampicin-resistant TB; unknown = absence of data regarding the resistance profile of the index patient.

Sex distribution remained stable throughout the project, with 53.2% women and 46.8% men. Most referrals (77.3%) were among household and close TB contacts, consistent with national prioritisation criteria. Although child contacts made up the largest subgroup (40.3%), the share of adult contacts increased substantially over time (from 4.6% in late 2021 to 19.8% in 2024), mirroring the broader expansion of testing to older age groups as programme capacity grew.

### QFT-Plus testing care cascade

Table 2 presents the testing care cascade by risk group and resistance profile of the index patient. Across all age groups of TB contacts, a DS-TB profile was the most common: 62.7%, 69.5%, 67.6%, and 70.9% among contacts under 5 years, aged 5–14, 15–17, and >18 years, respectively. Of the 11,495 individuals referred for testing, 9,788 (85.2%) completed testing. When stratified by risk group and index patient resistance profile (Table 2), the proportion of tested individuals was consistently above 85% in most categories, and often exceeded 90%, particularly among contacts of index patients with pre-XDR/XDR-TB. Of the individuals tested, 20.2% were positive overall. Positivity rates varied by risk group and resistance profile of the index patient (Table 2). Among TB contacts, the proportion of QFT-positive results increased with age: from 14.9% in children under 5 years to 28.1% in adults. Among all individuals with a positive test, 164 out of 1,978 (8.3%) were ultimately diagnosed with active TB, this was significantly lower in adults at 15/525 (*P* < 0.0001).

**Table 2.** QFT-Plus testing cascade in 17 regions in Ukraine by risk group and resistance profile of the index patient for the period October 2021–December 2024.

Risk groups	Resistance profile index patient	# referred for QFT testing	Proportion within the risk group	# tested	% tested	Positive QFT Result	% positive QFT results	# TB diagnosed (if QFT positive)	# TPT eligible (if QFT positive)	Started TPT (if QFT positive)	% of TPT eligible who are QFT positive that started TPT
TB contact children under 5 years	DS-TB	697	62.7%	564	80.9%	71	12.6%	6	65	60	92.3%
	H monoresistance	22	2.0%	17	77.3%	6	35.3%	2	4	4	100.0%
	RR/MDR	284	25.5%	241	84.9%	42	17.4%	2	40	29	72.5%
	Pre-XDR, XDR	66	5.9%	54	81.8%	14	25.9%	1	–	–	
	Unknown	43	3.9%	35	81.4%	3	8.6%	–	3	2	66.7%
	**Total**	**1,112**	100.0%	**911**	**81.9%**	**136**	**14.9%**	**11**	**112**	**95**	**84.8%**
TB contact children aged 5–14 years	DS-TB	3,220	69.5%	2,732	84.8%	526	19.3%	44	482	451	93.6%
	H monoresistance	71	1.5%	63	88.7%	19	30.2%	–	19	18	94.7%
	RR/MDR	922	19.9%	776	84.2%	219	28.2%	25	194	163	84.0%
	Pre-XDR, XDR	225	4.9%	212	94.2%	61	28.8%	6	–	–	
	Unknown	197	4.3%	160	81.2%	24	15.0%	2	22	22	100.0%
	**Total**	**4,635**	**100.0%**	**3,943**	**85.1%**	**849**	**21.5%**	**77**	**717**	**654**	**91.2%**
TB contact adolescent (15–17 years)	DS-TB	670	67.6%	573	85.5%	131	22.9%	14	117	110	94.0%
	H monoresistance	23	2.3%	22	95.7%	3	13.6%	–	3	3	100.0%
	RR/MDR	218	22.0%	198	90.8%	54	27.3%	7	47	35	74.5%
	Pre-XDR, XDR	51	5.1%	50	98.0%	7	14.0%	–	–	–	
	Unknown	29	2.9%	24	82.8%	5	20.8%	–	5	5	100.0%
	**Total**	**991**	**100.0%**	**867**	**87.5%**	**200**	**23.1%**	**21**	**172**	**153**	**89.0%**
TB contact adults	DS-TB	1,519	70.9%	1,330	87.6%	396	29.8%	12	384	357	93.0%
	H monoresistance	41	1.9%	37	90.2%	9	24.3%	–	9	7	77.8%
	RR/MDR	419	19.6%	354	84.5%	81	22.9%	1	80	69	86.3%
	Pre-XDR, XDR	70	3.3%	66	94.3%	21	31.8%	1	–	–	
	Unknown	93	4.3%	81	87.1%	18	22.2%	1	17	6	35.3%
	**Total**	**2,142**	**100.0%**	**1,868**	**87.2%**	**525**	**28.1%**	**15**	**490**	**439**	**89.6%**
Medical worker	**Total**	**340**	**100.0%**	**302**	**88.8%**	**42**	**13.9%**	**2**	**38**	**24**	**63.2%**
Individual with other reasons for examination	**Total**	**2,275**	**100.0%**	**1,897**	**83.4%**	**226**	**11.9%**	**38**	**188**	**147**	**78.2%**
Grand total	**Overall**	**11,495**	**100.0%**	**9,788**	**85.2%**	**1,978**	**20.2%**	**164**	**1,717**	**1,512**	**88.1%**

DS-TB = drug-susceptible TB; H monoresistance = isoniazid-resistant TB; pre-XDR, XDR = pre-extensively drug-resistant TB and extensively drug-resistant TB; QFT-Plus = QuantiFERON-TB Gold Plus test; RR/MDR = rifampicin-resistant/multidrug-resistant TB; unknown = absence of data regarding the resistance profile of the index patient.

Among those with a positive test and excluding individuals in whom active TB was diagnosed or contacts of pre-XDR/XDR-TB index patients, 1,717 persons were eligible for TPT and 1,512 (88.1%) initiated TPT. Initiation was highest among TB contacts aged 5–14 years at 91.2%, followed closely by adolescents (89.0%) and adult contacts (89.6%). TPT initiation was lowest among medical workers (63.2%) and referrals for other medical reasons (78.2%). TPT initiation varied by drug-resistance profile of the index patient, with the highest rate observed among DS-TB contacts and those with isoniazid (INH) monoresistance.

Table 3 outlines the care cascade by year, age group, sex, and region across all individuals. The number referred for testing increased substantially over time, from 2,773 in 2022 to 3,914 in 2024. The proportion of referred individuals tested remained consistent at around 85%; this was slightly lower among children under 5 years at 81.8% compared to 88.0% among adults. Proportions were similar by sex, 85.0% for men and 85.3% for women. Regional variation was observed. The proportion of QFT-Plus positives remained stable at approximately 20%. Among those QFT-Plus positive who were eligible for TPT, overall initiation was 88.1%, reaching a peak of 91.7% in 2024. By age, TPT initiation was highest (90.9%) among children aged 5–14 years and lowest among adults aged 18 years and older (85.1%). Regional variation was also notable, with some regions achieving 100% and others showing lower TPT initiation rates at 60%–70%. Details for those with other reasons for examination can be found in the supplementary data.

**Table 3.** QFT-Plus testing cascade in 17 regions in Ukraine by year, age group, sex, and region for the period October 2021–December 2024.

Grouping	# referred for QFT testing	Proportion within the group	# tested	% tested	Positive QFT result	% positive QFT results	# TB Diagnosed (if QFT positive)	# TPT eligible (if QFT positive)	Started TPT (if QFT positive)	% of TPT eligible who are QFT positive that started TPT
Year										
2021 (Oct–Dec)	1,084	9.4%	945	87.2%	183	19.4%	9	166	149	89.8%
2022 (Jan–Dec)	2,773	24.1%	2,328	84.0%	477	20.5%	47	403	326	80.9%
2023 (Jan–Dec)	3,724	32.4%	3,193	85.7%	600	18.8%	66	499	442	88.6%
2024 (Jan–Dec)	3,914	34.0%	3,322	84.9%	718	21.6%	42	649	595	91.7%
Age group										
0–4 years	1,359	11.8%	1,112	81.8%	158	14.2%	13	132	113	85.6%
5–14 years	5,948	51.7%	5,012	84.3%	939	18.7%	98	787	715	90.9%
15–17 years	1,179	10.3%	1,017	86.3%	207	20.4%	25	174	153	87.9%
18+ years	3,009	26.2%	2,647	88.0%	674	25.5%	28	624	531	85.1%
Sex										
Male	5,379	46.8%	4,571	85.0%	901	19.7%	85	763	677	88.7%
Female	6,116	53.2%	5,217	85.3%	1,077	20.6%	79	954	835	87.5%
Region (Oblast)										
Cherkasy	590	5.1%	526	89.2%	57	10.8%	1	50	47	94.0%
Chernihiv	610	5.3%	599	98.2%	85	14.2%	16	61	55	90.2%
Chernivtsi	677	5.9%	631	93.2%	136	21.6%	1	125	125	100.0%
Dnipro	887	7.7%	759	85.6%	151	19.9%	18	122	99	81.1%
Donetsk	130	1.1%	119	91.5%	19	16.0%	0	17	10	58.8%
Kherson	96	0.8%	84	87.5%	25	29.8%	0	22	15	68.2%
Kirovohrad	1,163	10.1%	1,131	97.2%	238	21.0%	70	160	157	98.1%
Kyiv	508	4.4%	420	82.7%	82	19.5%	1	76	71	93.4%
Lviv	1,076	9.4%	805	74.8%	205	25.5%	1	195	167	85.6%
Mykolaiv	191	1.7%	178	93.2%	60	33.7%	0	59	56	94.9%
Odesa	2,401	20.9%	1,644	68.5%	262	15.9%	3	255	211	82.7%
Poltava	907	7.9%	858	94.6%	168	19.6%	30	129	108	83.7%
Rivne	623	5.4%	540	86.7%	118	21.9%	0	114	113	99.1%
Vinnytsia	186	1.6%	171	91.9%	33	19.3%	0	32	32	100.0%
Volyn	1,037	9.0%	960	92.6%	203	21.1%	19	182	138	75.8%
Zakarpattia	302	2.6%	260	86.1%	96	36.9%	0	82	73	89.0%
Zaporizhzhia	111	1.0%	103	92.8%	40	38.8%	4	36	35	97.2%
Overall	11,495	100.0%	9,788	85.2%	1978	20.2%	164	1717	1,512	88.1%

QFT-Plus = QuantiFERON-TB Gold Plus test; TPT = TB preventive treatment.

From October 2021 to December 2024, a total of 421 individuals with negative QFT-Plus results initiated TPT. Of them, 188 (44.6%) were children under 5 years of age who are eligible for TPT irrespective of test results. Among others, the proportion receiving TPT despite a negative test result declined substantially over the project period – from 19.1% in Q4 2021 to 3.6% in 2024, indicating a favourable trend.

### TPT regimens

**Figure 1.**
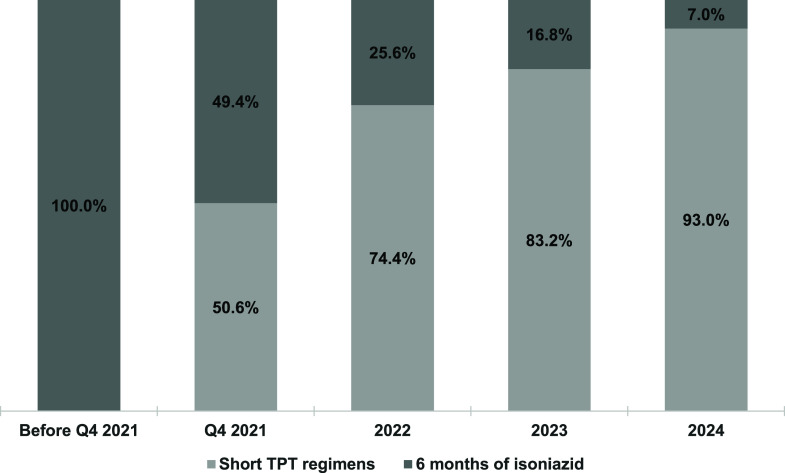
Trend in short- and long-term TB preventive treatment (TPT) regimens among individuals initiating preventive treatment for drug-sensitive TB, October 2021–December 2024. Q = quarter.

**Figure 2.**
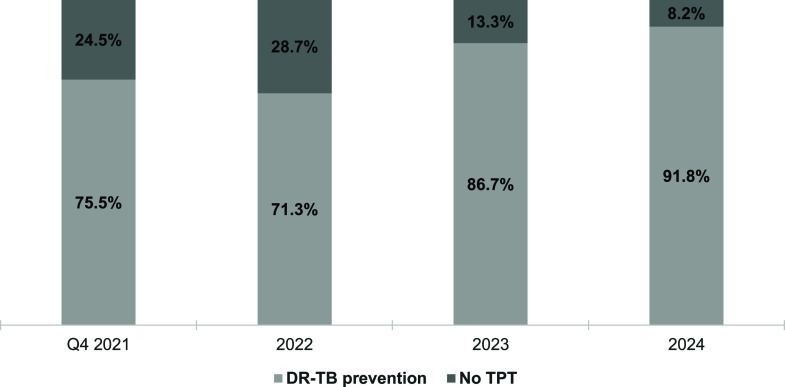
TB preventive treatment (TPT) initiation among drug-resistant TB (DR-TB) contacts, October 2021–December 2024. Q = quarter.

Among individuals initiating TPT for DS-TB, the distribution of short-term versus long-term regimens (Figure 1) shows that short-term regimens accounted for 50.6% of TPT prescriptions by the fourth quarter of 2021, increasing steadily to 93.0% in 2024. Among eligible DS-TB contacts, TPT initiation was high, with 978/1,048 (93.2%) individuals starting treatment between October 2021 and December 2024. For DR-TB, TPT was indicated for 396 contacts of DR-TB of whom 328 (82.8%) initiated treatment between October 2021 and December 2024, a significantly lower proportion than among DS-TB contacts (*P* < 0.001). However, the proportion initiating TPT increased from 75.5% (40/53) in the last quarter of 2021 to 91.8% (78/85) in 2024 (Figure 2).

## DISCUSSION

This analysis presents operational experiences from the STBCEU project, which supported the scale-up of QFT-Plus testing and TPT across 17 regions in Ukraine from 2021 to 2024. High uptake of QFT-Plus testing was achieved, with 85% of referred individuals completing testing, demonstrating feasibility across diverse and fragile settings. Losses of 15% along the care cascade highlight the need for strengthened patient support and active follow-up, consistent with the WHO Clinical Standards for TB Infection.^5^ Lower uptake among health care workers likely reflects programme prioritisation under constrained test availability and persistent scepticism regarding the value of TB infection testing in this group – barriers also documented internationally^5^ and requiring targeted operational strategies. Adult TB contacts demonstrated the highest QFT-Plus positivity, reinforcing the importance of expanding systematic screening beyond children and adolescents, in line with evidence from Lesotho, South Africa, and Tanzania.^13^

Linkage from diagnosis to prevention was strong, with TPT initiated in 93% of eligible DS-TB contacts and 83% of DR-TB contacts. Nonetheless, the absence of validated preventive regimens for contacts of pre-XDR/XDR-TB patients remains a critical gap recognised in global clinical standards^5^ and TB elimination frameworks.^14^ Until new evidence emerges, programmes should prioritise structured clinical follow-up, symptom monitoring, and timely evaluation for early disease detection while preparing for rapid adoption of emerging recommendations from ongoing DR-TB prevention trials.^12,15,16^

The programme successfully transitioned from 6-month INH regimens to shorter, patient-friendly options, including rifapentine-based TPT and levofloxacin for drug-resistant contacts. Evidence from the TB-CHAMP trial^17,18^ demonstrates high adherence and ≥80% regimen completion for levofloxacin among children exposed to MDR-TB, supporting feasibility even in complex settings and aligning with global priorities to improve care cascade completion.

A public–private collaborative model was a key enabler of IGRA scale-up. Private laboratories were contracted and reimbursed per completed valid test based on monthly reporting, ensuring payment only for delivered services and supporting consistent regional availability and timely reporting. Collaboration between private laboratories and TB facility staff strengthened diagnostic quality and linkage to care, in line with experiences from other settings.

This operational analysis has limitations. Conducted under routine programme conditions, it reflects real-world implementation but may be subject to data-quality constraints, including potential duplicate records and incomplete outcome data. Routine TST data were unavailable, treatment completion and safety outcomes could not yet be analysed, and population-level testing coverage could not be estimated due to the absence of denominator data. These outcomes will be reported separately once follow-up is complete.

## CONCLUSION

The STBCEU project demonstrated that targeted QFT-Plus testing and appropriate TPT regimens can be successfully operationalised using a public–private collaborative model in a fragile, high-burden, and partially conflict-affected setting. The intervention expanded TB infection diagnosis, improved access for previously underserved groups – particularly adult TB contacts – and supported the first national-scale implementation of DR-TB preventive treatment in Ukraine, directly addressing the project’s objectives of scaling up IGRA testing, increasing TPT uptake, and generating programmatic insights for national implementation. Important gaps remain. Until new evidence and guidance become available, programmes should prioritise structured clinical monitoring and rapid symptom evaluation, while preparing for timely adoption of emerging findings from ongoing global trials. Attrition along the testing cascade highlights the need to better understand and address operational and behavioural barriers to access. Future research should focus on treatment completion and adherence, interventions to reduce cascade losses, and the feasibility of preventive treatment for contacts exposed to highly drug-resistant TB strains. Continued investment in diagnostics, patient-centred support, and programme supervision will be essential to sustain and expand these gains.

## Supplementary Material
