# Supplementary material for: Experiences from Ukraine in expanding TB infection diagnosis and treatment, including for drug-resistant TB
Source: IJTLD Open. 2026 Apr 13;3(4):247–54. doi: 10.5588/ijtldopen.25.0670 (PMC13080308; doi:10.5588/ijtldopen.25.0670)
Supplement: Supplementary file 1 [file ijtldopen25-0670_supplementarydata1.pdf]

Supplement

Screening algorithm

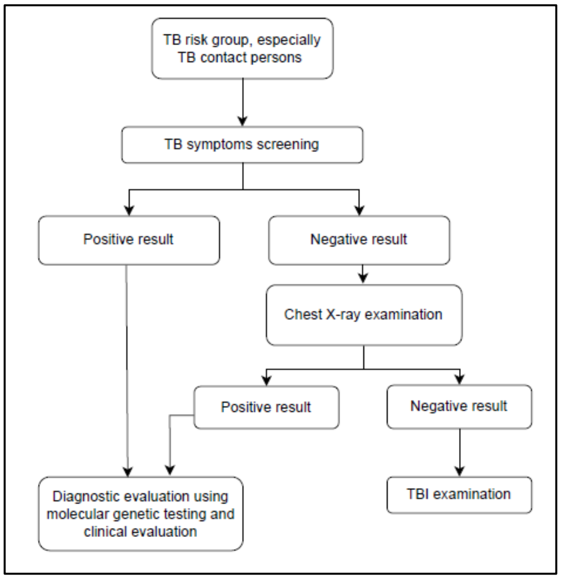

Figure S1. Algorithm for identifying individuals eligible for TB infection testing.

TB care cascade for group “Other reasons for examination”

Between October 2021 and December 2024, 2,275/11,495 (19.8%) individuals with reasons for examination other than TB contact or medical worker were referred for TBI diagnosis. This group consisted of 12 subgroups (see Table S1).

Over 91% of persons referred for testing were from the following 5 subgroups: persons with diseases that lead to immune suppression (32.7%), internally displaced persons (20.8%), children with positive/hyperergic TST result (19.3%), unspecified indication (10.4%), and individuals with fibrotic lung changes who have not received TB treatment (7.9%). QFT-Plus positivity varied by group. The highest rates of positivity were in the military personnel group (N=35, 42.9%), followed by individuals with fibrotic lung changes who have not received TB treatment (N=159, 23.3%), children with positive/hyperergic TST result (N=393,16.0%), and persons living with HIV (N=52, 15.4%). The overall level of TPT initiation was 78.2%. This was highest in children with positive/hyperergic TST result (95.3%), followed by persons with diseases that lead to immune suppression (90.9%). This was lower in military personnel (71.4%), internally displaced persons (70%), and PLHIV (62.5%).

Table S1. QFT-Plus testing cascade in 17 regions in Ukraine for group "Other reasons for examination" for the period October 2021–December 2024.

Grouping	# Referred for QFT testing	Proportion within the "other" group	# Tested	% Tested	# Positive QFT Result	% Positive QFT results	# TB Diagnosed	# TPT eligible (if QFT positive)	Started TPT (if QFT positive)	eligible who are QFT
Persons with diseases that lead to immune suppression	743	32.7%	664	89.4%	48	7.2%	4	44	40	90.9%
Internally displaced persons	473	20.8%	311	65.8%	21	6.8%	1	20	14	70.0%
Children with positive/hyperergic TST result	440	19.3%	393	89.3%	63	16.0%	20	43	41	95.3%
Unspecified indication	236	10.4%	202	85.6%	25	12.4%	-	25	11	44.0%
Individuals with fibrotic lung changes who have not received TB treatment	179	7.9%	159	88.8%	37	23.3%	12	25	17	68.0%
Children before BCG vaccination	84	3.7%	61	72.6%	7	11.5%	-	7	7	100.0%
Persons living with HIV	58	2.5%	52	89.7%	8	15.4%	-	8	5	62.5%
Military personnel	39	1.7%	35	89.7%	15	42.9%	1	14	10	71.4%
Persons living below the poverty line	16	0.7%	13	81.3%	1	7.7%	-	1	1	100.0%
Persons exposed to hazardous working conditions	5	0.2%	5	100.0%	1	20.0%	-	1	1	100.0%
Alcohol and drug-dependent individuals	1	0.0%	1	100.0%	-	0.0%	-	-	-	0.0%
Prisoners	1	0.0%	1	100.0%	-	0.0%	-	-	-	0.0%
**Total**	**2,275**	**100%**	**1,897**	**83.4%**	**226**	**11.9%**	**38**	**188**	**147**	**78.2%**

Abbreviations: BCG = Bacillus Calmette-Guérin; QFT-Plus = QuantiFERON-TB Gold Plus test; TB = tuberculosis; TPT = tuberculosis preventive treatment.
